# Ovarian Cancer: Tumor-Specific Urinary Micro-Peptides Profiling as Potential Biomarkers for Early Diagnosis

**DOI:** 10.3390/proteomes8040032

**Published:** 2020-10-29

**Authors:** Sulafa S. Murgan, Faisal J. Abd Elaziz, Abubakr M. A. Nasr, Mona E. E. Elfaki, Eltahir A. G. Khalil

**Affiliations:** 1 Department of Clinical Pathology and Immunology, Institute of Endemic Diseases, University of Khartoum, P. O. Box 45235, Khartoum 11111, Sudan; sulafamurgan@gmail.com; 2 Department of Obstetrics and Gynecology, Military Hospital, Omdurman 11111, Sudan; FaisalGalal@hotmail.com; 3 Department of Obstetrics and Gynecology, Faculty of Medicine, University of Khartoum, Khartoum 11111, Sudan; abubakrnasr@gmail.com; 4 Department of Microbiology, King Khalid University, Abha 62519, Saudi Arabia; melfaki@kku.edu.sa

**Keywords:** ovarian cancer, urinary micro-peptides, early diagnosis, Sudan

## Abstract

Ovarian cancer is the second major lethal gynecologic malignancy in developing countries. This study aimed to characterize urinary micro-peptides as potential diagnostic biomarkers for ovarian cancer. In a prospective, longitudinal and case-controlled study and following informed consent, urine and plasma samples were collected from 112 women with histologically-proven ovarian cancer and 200 apparently healthy age-matched volunteers. Urinary micro-peptides were detected and sequenced using SDS-PAGE and Edman degradation technique. Serum CA125 was detected in less than a quarter (23.2%, 26/112) of patients. One or more urinary micro-peptides were detected in about two thirds of the patients (62.5%, 70/112). A total of 40 patients had three bands (57.1%, 40/70), while two bands (15 and 35 kDa) were detected in 28.6% (20/70) of the patients. Isolated 45 kDa band was seen in 14.3% (10/70). No urinary micro-peptide was detected in the volunteers. The 15 and 35 kDa bands disappeared after 6 months of regular chemotherapy, while the 45 kDa band persisted in 2.9% (2/70) of the patients after treatment. The micro-peptides were identified as: Catalase (45 kDa), α-1 Acid Glycoprotein (35 kDa) and Peroxiredoxin-2 (15 kDa). Urinary catalase, α-1 Acid Glycoprotein and Peroxiredoxin-2 can be useful biomarkers for early detection and treatment response of ovarian cancer.

## 1. Introduction

Ovarian cancer is the seventh most common cancer and the most common cause of mortality from gynecological cancers worldwide. In developing countries, it ranks the second most common gynecological cancer and constitutes the fourth most common cancer in women. Limited reports from Sudan showed that ovarian cancer constitutes 6.8% of all recorded cancer, ranking as the sixth most common cancer for both sexes [1,2]. Ovarian cancer at its early stages (I/II) is difficult to diagnose until it spreads and advances to later stages (III/IV). This is because presenting symptoms are non-specific and are of limited discriminatory diagnostic value. Available diagnostic procedures are: medical history [pelvic examination], imaging [US, CT, MRI] and serological tests [CA-125] are of reduced sensitivity and specificity. Serum HCG, α-fetoprotein and lactate dehydrogenase (LDH) can be measured in young girls and adolescents with suspicion of ovarian tumors as complimentary tests to improve diagnostic accuracy. Cost is the main limiting factor for screening the whole postmenopausal population. In addition, most ovarian cancers grow rapidly and metastasize early, making it difficult to track location and progression. Routine screening of women for ovarian cancer is not recommended by any professional society, since no trial has shown improved survival for women undergoing screening. However, in some countries such as the UK, women who are likely to have an increased risk of ovarian cancer (family history of the disease) can be offered individual screening, although this will not necessarily detect the disease at an early stage [3,4,5]. Diagnosis at screening must be confirmed by surgery, fluid cytology and tissue biopsies for histopathology. Current researches focus on combining tumor markers [e.g., KLK6/7, GSTT1, PRSS8, FOLR1, ALDH1, miRNAs and proteomics…] with conventional tests to increase detection rates. The contribution of proteomics in cancer diagnosis is ever growing and could be of great diagnostic value in the near future [6,7,8,9,10,11,12,13]. 

This study aimed to identify and characterize candidate urinary micro-peptides as potential biomarkers for the early diagnosis of ovarian cancer.

## 2. Experimental Section

### 2.1. Ethical Considerations

The study protocol was reviewed and passed by the Ethics Committee of the Institute of Endemic Diseases, University of Khartoum. Written informed consents were obtained from enrolled patients and apparently healthy volunteers.

### 2.2. Study Design, Sites, and Duration

This was a prospective, longitudinal, case-controlled and hospital-based study that was conducted over two years (January 2014–December 2015) at tertiary-referred hospitals.

### 2.3. Study Population

A total of 112 women with histologically-confirmed ovarian cancers (cases) and 200 apparently healthy volunteers (comparators) were also enrolled. Follow-up was carried out every 6 months for a total of 2 years after ovarectomy and chemotherapy. 

### 2.4. Samples

Five mls of whole blood and morning urine samples were collected.

### 2.5. Determination of Tumor Biomarker CA125 Using ELISA Technique [GenAsia Biotech Co. Ltd, Shanghai, China]

Sera C125 levels were determined as described by the manufacturer. Briefly: Sera samples and standards were added with secondary antibody-labeled with biotin and buffer to plate wells. The mixture was incubated for 60 min at 37 °C, the plates were washed five times. Fifty μL of chromogen solutions were added to the plates and incubated for 10 min at 37 °C for color development, then 50 μL of stop solution were added. The developed color was read at 450 nm within 10 min, and CA125 levels were calculated from the constructed standard curve.

### 2.6. Polyacrylamide Gel -SDS Gel Electrophoresis (PAGE-SDS Electrophoresis)

#### Urine Protein Precipitation

Urine ultra-centrifugation at 2000× *g* for 20–30 min at room temperature was done within 1 h of collection. A total of 400 μL of Acetonitrile (CAN) [containing 0.1% Tri-Fluoroacetic acid, TFA] was added to 200 μL of urine samples, and acetone precipitation was followed by vigorous vortexing for 5 s and allowed to stand at room temperature for 30 min. Samples were then spun for 10 min at 12,000 rpm, and the deposit was aliquoted and stored for subsequent analysis. The urinary deposits were subjected to resolving gel electrophoresis. Separation of the proteins was carried out at a constant voltage of 100V for one and a half hour. The resolving gel was then transferred to the staining solution for 24 h at room temperature. The resolving gel was then de-stained and photographed. Bands with known sizes were included for reference.

### 2.7. Sequencing and Identification of Selected Urine Peptides

Selected urine peptides were cut from polyacrylamide-SDS gel using sterile blades and placed inside sterile plastic Eppendorf tubes labeled according to the size of bands. The samples were sent for commercial sequencing using Edman Degradation technique [The Biochemistry Laboratory, Sangon Biotech Co. Ltd, Shanghai, China].

The Edman degradation technique is briefly as follows:

Coupling: the N-terminus of the protein couples with Phenyl-isothiocyanate (PITC) under basic conditions to form a phenylthio-carbamyl (PTC)-polypeptide.

Cleavage: the peptide bond of the N-terminal PTC-residue undergoes acid cleavage from the polypeptide chain. This results in the release of an unstable aniline-thiazolinone (ATZ) derivative of the amino acid. Conversion: the unstable ATZ-amino acid is converted into the corresponding phenylthiohydantoin (PTH) derivative. The PTH-amino acid is stable. At the end of each cycle of degradation, the PTH-amino acid is separated from the reaction by-products and identified by High-Pressure Liquid Chromatography (HPLC) and UV absorbance, respectively. 

Searches for homologous sequences to the isolated micropeptides were performed by BLAST (Basic Local Alignment Search Tool), by comparing identified primary sequence of amino-acid to different proteins sequences [14,15].

### 2.8. Statistical Analysis

The collected data was analyzed using SPSS version 16; frequencies, *t*-test, correlation and significance for SDS_PAGE bands were analyzed. The frequencies reported for CA 125 were compared to presence of urinary micro-peptides. Micro-peptides band patterns were compared between comparator and patients. The sensitivity, specificity, positive and negative predictive values were calculated for CA125 antigen and urine micro-peptides tests. *p* value of <0.05 was considered statistically significant.

## 3. Results

The mean age of the study patients was 46.5 ± 28 years, while that of apparently healthy volunteers was 42.5 ± 23 years. The majority of patients [90%, 101/112] were within the age group 30–70 years (*p* = 0.01). Most patients (>90%) reported non-specific symptoms (e.g., abdominal discomfort and pelvic pain), while irregular vaginal bleeding was reported by ~1% of patients. Most patients (88/112, 78.6%) had symptoms of ≤12 months duration. Married women with children were less significantly affected (23/112, 20.5%) compared to those married with no children or single women (89/112, 79.5%) (*p* = 0.0001). A minority of patients (6/112, 5.4%) had concurrent malignancies (colon cancer/breast cancer). The majority of patients (93.8%, 105/112) had advanced disease (Stage III-V). Histologically, serous adenocarcinoma was seen in the majority (81.2%, 91/112) of the study patients and it was seen in more than half (58.1%) of patients with advanced disease. Mucinous types were seen in 10.7% (12/112) of patients, while endometroid and poorly differentiated types were seen in a minority (5.4%, 6/112 and 2.7%, 3/112, respectively) (Table 1). 

**Figure 1:**
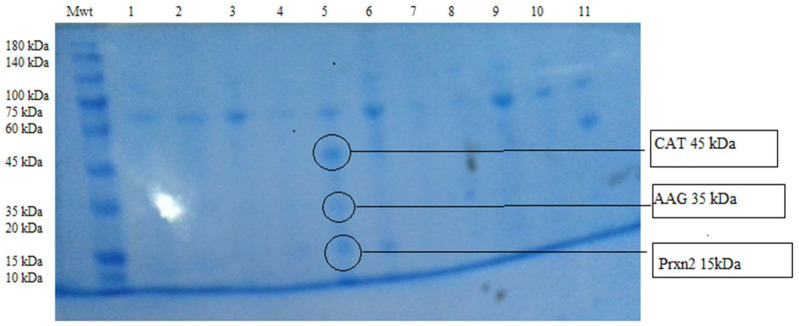
SDS 12.5% polyacrylamide gel electrophoresis [SDS-PAGE] gel. Mwt: Protein ladder. Lanes 1, 2: Apparently healthy volunteers. Lanes 3, 4, 5, 6, 7, 8,9,10 and 11 Ovarian Cancer study cases. Lane 5 [three bands: size 45, 35, 15 kDa] Lane 6 [15, 45 kDa bands]. Lane 8 [35 kDa band].

One or more urinary micro-peptides were detected in about two thirds of the patients (62.5%, 70/112), they were more frequent (64.3%) in patients with advanced disease (Stage III/IV) compared to those with early disease (35.7%). A total of 40 patients had three bands (57.1%, 40/70), two bands (15 and 35 kDa) were detected in 28.6% (20/70) of the patients, while an isolated 45 kDa band was seen in 14.3% (10/70). Urinary micro-peptides were detected with different frequencies in different disease stages: stage I (7/70, 10%), stage II (18/70, 25.7%), stage III (17/70, 24.3%), and in stage IV (28/70, 40%) with highest frequency at stage IV. No urinary micro-peptide band was detected in apparently healthy volunteers. The 15 kDa and 35 kDa bands disappeared after 6 months of chemotherapy and follow up, while the 45 kDa remained in 2.9% (2/70) of the patients at 2 years follow up (Figure 1).

The N-terminal sequence of the 45kDa micro-peptide was found to be:

**MWDVSTGMCLMTGVGHDNWVR** [Methionine, Tryptophan, Aspartate, Valine, Serine, Threonine, Glycine, Methionine, Cysteine, Leucine, Methionine, Threonine, Glycine, Valine, Glycine, Histidine, Aspartate, Asparagine, Tryptophan, Valine, Arginine], which is 95% homologous to the Human erythrocyte Catalase. The sequence of the 35 kDa micro-peptide was **RYVGGQEHFAHLLILRD** [Arginine Tyrosine Valine Glycine Glycine Glutamine Glutamate Histidine Phenylalanine Alanine Histidine Leucine Leucine Isoleucine Leucine Arginine Aspartate], and was 100% homologus to human α1-Acid Glycoprotein. The micro-peptide band size 15 kDa was sequenced as **TVAAPSVFIFPPSDEQLK** [Threonine Valine Alanine Alanine Proline Serine Valine Phenylalanine Isoleucine Phenylalanine Proline Proline Serine Aspartate Glutamate Glutamine Leucine Lysine] and 100% homologous to human Peroxiredoxin-2.

CA125 was detected in the plasma of about a quarter of patients (23.2%, 26/112). CA125 reactivity was not significantly different in different age groups [60–70 years, 50-59 years, 40–49 years, and 30–39 years were 8.0%, 5.4%, 5.4%, and 4.5%, respectively, *p* ˃ 0.5]. A quarter (23.2%, 6/26) of patients with CA125 reactivity had urinary peptides. CA125 antigen disappeared in 42.3% (11/26) at 6 months of chemotherapy. CA125 antigen was not detected in the sera of the apparently healthy volunteers. The sensitivity and specificity of CA125 test were calculated as 23.2% and 100%, respectively, while the positive and negative predictive values were 100% and 69.9%, respectively. While the sensitivity and specificity of urinary micro-peptides was 62.5% and 100%, respectively, with positive and negative predictive values of 100% and 82.6%, respectively.

## 4. Discussion

Understanding the role of proteomes/micro-peptides in human cancers together with their pharmacokinetics can greatly help in devising new diagnostic tests and therapeutics. It has been shown that Catalase antioxidant enzyme enhances the survival of detached breast/ovarian cancer cells and helps their successful metastasis [16]. Likewise, α-1 Acid Glycoprotein has been shown to increase in the plasma of patients with ovarian, hepatic, lung, and gastric cancers with probable roles in modulating cancer immune responses and cancer progression [17]. On the other hand, peroxiredoxins are peroxidases that are involved in the peroxide reduction of H_2_O_2_ and peroxynitrite. These enzymes are pivotal for the control of cell growth/differentiation, immune response, and apoptosis, with accumulating evidence of their involvement in carcinogenesis [18]. Secretion of these three micro-peptides in the urine of patients with ovarian cancer makes them legitimate targets for development of diagnostics and therapeutics.

Ovarian cancer patients in Sudan were mostly of the age group 60–70 years with most patients reporting vague and non-specific symptoms as has been previously reported in the African region and globally [19,20]. Serous adenocarcinomas were seen in the majority of our patients with advanced disease in more than half, while other histological types were seen in the minority in agreement with previous reports [21,22]. The low diagnostic sensitivity, the high specificity and the high negative predictive value of plasma CA125 test were confirmed in this study. On the other hand, urinary micro-peptides were detected in a considerable number of our patients, with a steady increase to reach highest frequency in stage IV. The moderately high frequencies in stage I/II make urinary micro-peptides a moderately sensitive, highly specific, and highly predictive early diagnostic parameter. No urinary micro-peptides were detected among apparently healthy volunteers, probably indicating their uniqueness to cancer tissues. These micro-peptides can be incorporated in a dip-stick test that can be simple, non-invasive, and cheap to be applied within a nation-wide screening program. This will overcome the cost-limiting factor of available screening tests. It is not clear why only a quarter of cases with C125 reactivity showed urinary micro-peptides reactivity. This is probably due to the fact that different molecules are expressed differently by the same tumor. Recently, Whitwell and colleagues claimed significant improvement in CA125 diagnostic accuracy using longitudinal multi-marker model for early detection of ovarian cancer [23]. A decline in expression of CA125 during chemotherapy which is considered a favorable prognostic sign was confirmed in this study in agreement with previous reports [10]. The disappearance of 15 and 35 kDa bands in our patients can be a useful indicator of response to treatment.

It is evident from this study that combining clinical data, CA125 (multi-marker models), imaging, and urinary proteomics can markedly improve early detection of ovarian cancer in women at risk (aged 60–70 years). The reduction/disappearance of urinary micro-peptides can prove a helpful prognostic and follow up indicator. The increased negative predictive value of urinary micro-peptides compared to CA 125 will increase the number of cases in screening programs [24,25].

In conclusion, urinary micro-peptides [Catalase, α-1 Acid Glycoprotein and Peroxiredoxin-2] can be useful biomarkers for early detection and treatment response monitoring of ovarian cancer. Combining the CA125 test, imaging, and urinary micro-peptides can markedly improve the early diagnosis of ovarian cancer.

## Figures and Tables

**Table 1:** Demographic, clinical and laboratory findings of the study population.

Study Groups:	Age (yrs ± SD)				Presentation		Disease Stage	
				*Abdominal*	*Pelvic*	*Vaginal*	*Stage I/II*	*Stage III/IV*
				*Discomfort*	*pain*	*bleeding*		
**Apparently healthy**	**46.5 ± 28**			**Nil**	**Nil**	**Nil**		
*(n=200)*								
**Study patients**	**42.5 ± 23**			**>90%**	**>90%**	**1%**	**34/112**	**78/112**
*(n = 112)*							*−30.40%*	*−69.40%*
**Urinary micro-peptides:**							**+ve**	**+ve**
*(Patients with urinary peptides = 70)*							**10%/25.7%**	**24.3%/40%**
**Patients with Urinary peptides and C125 reactivity:**						**6/26 *(23.2%)***		
**Histological Types:**		**Sero adenocarcinoma**	**mucinous adenocarcinoma**		**other types**			
		**91/112** *(81.2%)*	**11/112** *(9.8%)*		**10/122** *(9.0%)*			

The underline, italics and bold words are used to highlight content.
